# Supplementary material for: Approximating multivariate posterior distribution functions from Monte Carlo samples for sequential Bayesian inference
Source: PLoS One. 2020 Mar 13;15(3):e0230101. doi: 10.1371/journal.pone.0230101 (PMC7069631; doi:10.1371/journal.pone.0230101)
Supplement: Attachment — Submitted filename: reply to reviewers_v2.docx [file pone.0230101.s002.docx]

Reviewer #1: I enjoyed reading this manuscript, which is written very clearly and focuses on a simple idea using existing tools. The emphasis is primarily on splitting a dataset and then running posterior sampling on the first part of the dataset, obtaining an analytic approximation of the posterior and using this as the prior in the analysis of the second part of the dataset. I do have some major concerns and comments:

*We’d like to thank the reviewer for the positive words; we will reply to the concerns and comments below*. *The reviewer comments are in normal font and our replies are printed in italics.*

(1) The problem of scalable computation for large datasets is quite different from the problem of analyzing multiple datasets in that it is important to account for differences across the datasets in the multiple dataset case. There may be heterogeneity in the parameters which crucial needs to be taken into account and there is a rich literature on associated hierarchical models and methods for meta analysis. This type of issue is not discussed at all in the paper, and I would worry that the naive reviewer may use the proposed method not realizing that it is effectively the same as pooling the different datasets together and using a single statistical model which assumed observations from different datasets are exchangeable. This needs to be properly addressed.

*We agree with the reviewer that performing statistical inference with multiple datasets may require a model that accounts for differences between datasets, such as by using a hierarchical model or by having dataset-specific parameters. The sequential inference approach we have taken is equally applicable whether the model under consideration incorporates a hierarchical structure/dataset-specific parameters or not. Indeed, in our Lotka-Volterra example the model has 5 global parameters and 2 dataset-specific parameters. To further clarify this and hopefully prevent naïve use, we have added several sentences (page 2, lines 32-35) to emphasize that hierarchical models may be necessary when modeling multiple datasets.*

(2) If the focus is instead entirely on accelerating computation for a single large dataset by splitting the data into smaller datasets that are analyzed separately in a sequential manner, then there is a large relevant literature that needs to be properly discussed. Currently the authors just cite a single article based on using a product equation with kernel approximations plugged in - this method they cite actually has quite poor performance relative to others as discussed in more recent publications in this area. One needs to motivate why analyze the data sequentially instead of in an embarrassingly parallel manner, which would seem to be faster. Hence, the practical motivation & support relative to the literature for the method is quite lacking.

*We see now that the motivation of the manuscript was not entirely clearly described. The main point we wanted to investigate was whether sequential inference with Monte Carlo sampling can give accurate results at all; and if it does, if it allows inference to be faster than doing the joint inference. We did describe this in the abstract: “Here, we investigated to what extent we can obtain an accurate joint posterior from two datasets if the inference is done sequentially rather than jointly, under the condition that each inference step is done using Monte Carlo sampling.”. But the motivation was not clear from the introduction, therefore we have now added a paragraph to the introduction to further motivate our approach and clarify our reasoning. So, to be clear, our focus is not simply on accelerating computation by following a divide-and-conquer strategy on a single data set.*

*Regarding computational advances, we actually found that sequential inference is not faster than joint inference - rather the opposite. In the results section “Efficiency of sequential versus joint inference” we show that joint inference is faster than sequential inference as judged by the accuracy obtained by a fixed number of model evaluation. We also mention this result in the abstract. We had hoped that sequential inference might be faster, and in that sense the results are not what we had hoped for. Nevertheless we believe that these results are still worthwhile to publish, as others may be attempting similar approaches, and we still believe sequential inference can have conceptual and practical advantages.*

(3) A very very major issue that is not properly discussed in my view is the curse of dimensionality and the fact that the proposed methods are not going to do well at all when the number of parameters in the model is not quite small - say about 5 with 10 being too large most likely. Most interesting Bayesian models have many-many more parameters than this & this limits the scope of the methods. The problem is that the posterior approximation methods break down rapidly in dimensionality and the authors really don’t consider methods that are particularly scalable in terms of density estimation - e.g., mixtures of factor analyzers and such; see “Geometric density estimation” work by Wang, Canale and Dunson for example. One seemingly big motivation for not taking a sequential approach is that one can run posterior computation in parallel for chunks of the data & then combine in a targeted manner for low-dimensional functionals and parameters of interest instead of combining for the entire high-dimensional posterior; see, for example, Li, Srivastava and Dunson 2017 Biometrika.

*We’d like to thank the reviewer for the suggestion to look into mixtures of factor analyzers. We have tested them, and they indeed perform very well, particularly when there are ridges in the posterior. We have now included mixtures of factor analyzers in all the analyses in the paper and in the software package.*

*We also investigated geometric density estimation (GEODE) from Wang, Canale and Dunson. This method is focused on situations of much higher dimensions (up to hundreds of thousands) and GEODE by itself did not perform well in our setting. Given our focus on lower-dimensional problems, we therefore think that including GEODE in our paper would be unfair. The GEODE paper also describes mixtures of GEODEs, which will probably do better than a single GEODE in our cases, however, we could not find an (R) implementation for fitting mixtures of GEODEs and it is not straightforward to implement ourselves.*

*We completely agree with the reviewer that the curse of dimensionality is an important problem in statistical modeling. In the present paper we have focused on lower numbers of dimensions (up to 10). The reason for this was that it seemed useful to us to look at a more manageable case with lower dimensions first and thoroughly explore that before attempting the (probably) more difficult higher dimensional setting. We do look explicitly at the effect of increasing dimensionality in the theoretical examples, where we could see that e.g. Gaussian processes are better in lower dimensions whereas e.g. Gaussian mixture become better in higher dimensions. We have added a discussion of this in the discussion section.*

*Importantly, there are also many applications of applied Bayesian analysis with lower-dimensional models. For example, in gravitational wave analysis, models with 9-15 parameters are of primary interest (Veitch et al, Parameter estimation for compact binaries with ground-based gravitational-wave observations using LALInference); and these inference tasks are far from trivial and require days of MCMC sampling, showing that inference methods for lower dimensions settings are also of interest.*

(4) If you do have a setting with very small numbers of parameters in the model and very large sample size (motivating the proposed approach) then it would seem that simpler methods are well justified - for example, Laplace approximation and Gaussian approximations to the posterior would be accurate.

*In the limit of infinite data (and with a number of important assumptions), a Gaussian posterior should indeed be accurate. But in practice, posterior distributions of models with few parameters can be strongly non-Gaussian despite a large amount of data. For example, in [Girolami M, Bayesian inference for differential equations, Theoretical Computer Science 2008] an example is given of a simulation experiment with a strongly non-Gaussian posterior of a two-parameter model, despite 120 data points – already more data than might typically be available. We also included a single Gaussian approximation in our experiments, and in all cases a single Gaussian is outperformed by a Gaussian mixture or some of the other approximation methods.*

Hence, I’m wondering if there is any real motivation for the proposed approach relative to the literature.

*We hope that we have now better explained the motivation for the paper – namely that there are various cases in which sequential inference can be useful or appealing; and that there is use for inference methods also in lower-dimensional settings. Approximating posterior distributions from Monte Carlo samples is not trivial, and to the best of our knowledge there are few comparisons of different estimation methods in this context. By investigating a number of theoretical examples and two applications we outlined where challenges lie and where approximation methods differ. We believe these analyses can be beneficial for others who might attempt similar approaches.*

Reviewer #2: In the manuscript "Approximating multivariate posterior distribution functions from Monte Carlo samples for sequential Bayesian inference", Bram Thijssen and co-workers present an evaluation of sequential inference in the context of Bayesian inference. This manuscript evaluates different methods for the approximation of posterior distributions such as kernel density estimation, Gaussian mixtures and vine copulas. The authors test the results of the approaches on two examples with real datasets, and compared to classical joint inference.

The topic of Bayesian inference is very relevant and there are many open questions. Bayesian inference is often achieved with Monte Carlo sampling approaches. One of the key problems is that Monte Carlo sampling can be very computationally demanding for complex models, therefore methods to reduce this bottleneck are needed. In this manuscript, the authors evaluate this problem from the experimental data perspective by assessing different methods for sequential estimation of the posterior distribution from the Monte Carlo samples of consecutive and independent datasets.

The key contributions are in my opinion (1) collecting and testing many methods for estimating a functional approximation of the posterior distribution and (2) the development of a reusable open-source toolbox/library. This evaluation is in my opinion a valuable contribution. I appreciate that the authors provide reusable code, but I have to admit that I did not have the time to test it. To my mind, it is very valuable that the authors also discuss and demonstrate the limitations of the approaches.

*We’d like to thank the reviewer for the positive remarks. We will reply to the concerns and comments below*. *The reviewer comments are in normal font and our replies are printed in italics.*

======

MAJOR

======

- In the introduction the authors refer to the case of challenging Bayesian inference for complex models (line 8). What do the authors consider here as a "complex model"?

*In that sentence, a complex model would be anything with an analytically intractable posterior, so we changed the text to state that instead. In general, we consider a model complex if its posterior also has features which make sampling challenging, such as multimodality, pronounced ridges or valleys, or non-Gaussian tails.*

- It would be great if the authors could comment on the use of the methods for high-dimensional inference, e.g. Hug et al., High-dimensional Bayesian parameter estimation: case study for a model of JAK2/STAT5 signaling. Mathematical Biosciences, 246(2), 293-304, 2013.

*We have included a reference to this paper in the introduction as an example of how Bayesian inference can be challenging in practice. Hug et al also discusses profile likelihoods as alternative to fully Bayesian inference to tackle higher-dimensional problems, but we focus here on lower-dimensional problems (up to 10 dimensions).*

- The authors only present one model in which the results are successful, are there other examples that could be included? In particular additional artificial examples would in my opinion be helpful.

*We have now added two additional artificial examples to the paper, to highlight other complexities in posterior probabilities, specifically ridges and heavy tails (see Figure 3). We agree that it would be nice to also include further applied analyses, however the paper is already quite long at 21 pages without figures, and we believe the modest conclusions are in line with the amount of evidence provided. We’d like to emphasize that in the second example, splitting the data by observable also still works well and can therefore also be considered a successful example of sequential inference. Only when the inference is split by experiment sequential inference breaks down. So, from this perspective, there are two successful examples and one unsuccessful example in the manuscript.*

- The authors mention that "Estimating functional forms of posterior distributions ... is an established part of ... in the context of estimating orbital eccentricities in astronomy". Are there any other fields in which this is currently used? e.g. Eriksson et al., Uncertainty quantification, propagation and characterization by Bayesian analysis combined with global sensitivity analysis applied to dynamical intracellular pathway models, Bioinformatics, 35(2), 2019.

*We looked at the paper from Eriksson et al, but as far as we can see, they do not estimate functional forms of the posterior distributions. They do use sampler output for further sensitivity analysis, but they only use the samples directly rather than estimating functional forms, so we don’t think this could be used as an example here.*

- Between line 16 and 17: "In our modeling of biological systems" The authors mention the computational challenge they encountered regarding inference with multiple datasets. I think this is a very common challenge in the community, therefore it would be beneficial to cite additional examples (ideally from other groups) showing this.

*Also in reply to reviewer #1 we have changed the introduction to better describe the motivation for the sequential inference approach. We added several more references here of cases where Bayesian inference was computationally challenging.*

- I was missing the discussion of radial basis faction approximations for posterior approximations. This approach has already been applied for posterior approximation(e.g. Fröhlich et al., Radial Basis Function Approximations of Bayesian Parameter Posterior Densities for Uncertainty Analysis. In: Mendes P., Dada J.O., Smallbone K. (eds) Computational Methods in Systems Biology. CMSB 2014. Lecture Notes in Computer Science, vol 8859. Springer, Cham, 2014).

*To our knowledge, (Gaussian) radial basis functions are identical to Gaussian mixtures, see e.g. [Figueiredo M.A.T., On Gaussian radial basis function approximations: interpretation, extensions, and learning strategies] or [Miller and Uyar, Combined Learning and Use for a Mixture Model Equivalent to the RBF Classifier]. It then comes down to how exactly the mixture distribution or radial basis functions are fitted. Assuming each fitting procedure converges to a good solution there should not be any differences in the resulting approximation.*

- The performance of several of the approximation methods depend on many implementation details. For example, the authors list the R package VineCopula for the evaluation of VCs. What about the other methods? It would be valuable to clarify this in the text.

*We thank the reviewer for pointing this out - we tried to mention all the main choices and software packages for all the approximation methods, but we missed some important aspects. For vine copulas we relied on an external package, hence the citation. Gaussian mixtures and Gaussian processes are implemented directly in mvdens. We now added details on the EM for Gaussian mixtures and the optimization of the GP. For multivariate kernel density estimation, we used the package “ks” for which we now included a reference as well, and for the newly added mixtures of factor analyzers we use the package “EMMIXmfa”.*

- Line 312: As the authors know, it is beneficial to use log parameters instead of natural scale. Can the authors apply the bounded setting to log parameters with broader bounds instead of the natural scale?

*Although we could of course add this analysis to the paper, the manuscript is already quite long, and we’re not entirely sure what this would add to the paper. An unbounded-log space and bounded-natural space would seem to us to be the two relevant cases.*

- Can the authors provide a computation time comparison for the predator-prey model? Since this model works in the sequential setting, a comparison respect to the joint setting regarding computation time would be great.

*We indeed pondered how to best show a comparison of the efficiency of sequential inference. The difficulty is that sampling-based inference is always a trade-off between computation time and accuracy – more samples will lead to more accurate/stable results. Sequential inference can be faster, but it introduces an extra error due to the approximation. So directly comparing the time it takes isn’t really meaningful – it should then be compared to how long it takes to reach a similar accuracy. Or, the other way around, what is the accuracy achievable with a given amount of computation time? We think the latter is the best way to show a comparison of the efficiency, so we’ve included that in the paper (section on efficiency of sequential vs joint inference).*

*To give a comparison of the computation time of the different approximation methods, we included an extra column in table 2 with the typical time it takes to fit a 10-dimensional approximation for 1,000 samples. The computation times differs hugely between methods, partially because the implementations have different levels of code optimization, and partially because some methods indeed involve more extensive computation than others.*

- Line 191: In MCMC chain, individual samples are dependent. May I ask how this dependence structure (which is disregarded in the first example) will influence the performance of methods? Is it possible to account for it? How is this related to the required sample size?

*Autocorrelation of MCMC samples will indeed affect the accuracy of the functional approximations. The approximations themselves are still valid even if the samples are correlated, e.g. the mean and variance calculated over correlated samples are still valid estimators (by Birkhoff’s ergodic theorem), but increasing autocorrelation or a smaller number of samples increases the sampling error in the estimates. We verified this; and indeed for example a GMM approximation is even slightly better on 100,000 correlated samples than it is on the same samples but subsampled to 1,000 to reduce autocorrelation (this point has been described in the MCMC literature (see e.g. Link and Eaton, On thinning of chains in MCMC, Methods in Ecology and Evolution 2012)).*

*One point where autocorrelation could give issues is when using the BIC, as its derivation does include an assumption on samples being independent. However, we now switched to using the AIC in all cases anyway (see explanation below in reply to the question about AIC vc BIC), so this is no longer an issue.*

*Despite this, in the examples involving MCMC samples we used excessively long MCMC chains with subsequent thinning to ensure that the MCMC samples are essentially uncorrelated; we now mention this in the methods. Keeping all samples would be slightly better, but then fitting the approximation methods would take much longer.*

- Line 204: As the authors mention, it is not surprising that GM performs best.

I would find it important to test the method on other common topologies, including structures which arise of parameters are non identifiable (see e.g. Raue et al., Joining forces of Bayesian and frequentist methodology: A study for inference in the presence of non-identifiability. Philos T Roy Soc A, 2013).

*We have now included two additional theoretical examples to further investigate the performance of the different approximation methods with different features which can arise in posterior distributions; specifically ridges and heavy tails. This indeed further highlights where each of the approximation methods have their strengths – e.g. the newly added factor analyzers are better at approximating ridges; while all methods have difficulties with heavy tails in high dimensions (which fits with the results from the failure case where the tails are the problem). We’d like to note that some of the posteriors in the practical examples do already contain partial non-identifiability.*

======

MINOR

======

- Line 32: Maybe the authors can shortly mention why is challenging to meet the constraint (i.e. must integrate to one) as scaling should be always applicable.

*In order to ensure that the probability distribution integrates to one, scaling the regression function is only an option if we can calculate the scaling factor, and this requires the ability to efficiently integrate the regression function, which is not always feasible.*

- Line 79: Why for D > 4 only the authors only estimate a diagonal matrix? Has this been shown to perform better? What about bootstrapping methods for bandwidth selection instead of plug-in bandwidth?

*This cut-off was quite arbitrary. We have now increased this limit to 6, but it is still capped at 6 for the practical reason that the Hpi function in the ks package only supports full-covariance kernel density estimation up to 6 dimensions. For dimensions up to 6, we include both full-covariance and diagonal covariance KD’s in the analyses; full covariance matrices are better as one would hope. It would be great to be able to use higher-dimensional full-covariance KDs, but this likely requires specialized methods, such as the method described by Liu et al (Sparse Nonparametric Density Estimation in High Dimensions Using the Rodeo, AISTATS07), but unfortunately no implementation of that method is available.*

*When redoing the inference for the revision, we did notice that using the normal reference rule for diagonal matrices gives better performance than using the univariate plug-in bandwidths as before, so we now changed to the normal reference rule instead for the diagonal KD’s.*

- Line 214: I am not an expert in the field of copulas, but the authors mention that "the available copula functions are designed to describe the shape of a single model". Do exist any multi-modal copulas (even though not available in the R library)?

*We are also not aware of any method describing multi-modal copula functions. But somewhat related, there have at least been reports of using mixtures of copulas to describe multi-modal distributions using copulas (e.g. Tewari et al, Parametric Characterization of Multimodal Distributions with Non-Gaussian Modes); we have added a reference to this in the discussion as another possible avenue to explore.*

- Can the authors clarify why was BIC used for GM while AIC for VC?

*This was arbitrary and is indeed inconsistent. The AIC is more appropriate, so we have changed the analyses to use the AIC for all cases of model selection, including the selection of copulas, number of components in mixtures and now the number of factors in the factor analyzers.*

- Line 240: The split of parameter beta appears to introduce a structural non-identifiability. I would suggest that the authors mention and discuss this.

*If only the number of lynxes and hares would be available than it would indeed be non-identifiable. However, the hare dataset also has measurements of the number of offspring per hare, which depends only on beta_stress, hence this parameter is actually identifiable when the hare dataset is included.*

- Line 259: How realistic is the assumption of the noise levels? Has this been quantified? If not, I would suggest to estimate sigma along with the dynamic parameters.

*We fixed the sigma parameters to keep the number of variables low. Given (scaled) measurements which range from 0 to 1, a nosie level of 15% seems reasonable, and the posterior predictive plots in Figure 4 show a reasonable fit and level of confidence, in our opinion. In general, we agree with the reviewer that estimating the variance as additional unknown parameter would be a good thing to do, but we believe it is not so relevant for whether sequential inference works or not and we would like to keep the number of parameters low.*

- Line 271: Maybe the authors can integrate Fig. 5A-C better into the text.

*We thank the reviewer for this comment. Our description of this figure was indeed overly brief, We now elaborated more on the features of the posterior distribution.*

- Line 341: Could the authors clarify what are the differences between the different realizations? Different starting points, random seed ... ?

*The entire procedure is done anew in each run. This indeed includes different starting points, random seeds, setting the sampling hyperparamaters, etc. We’ve added this to the text.*

- Line 348: For me is not clear to spot the slight bias in \beta_{kill} when the hare dataset is used first that the authors mention (Fig. 8). Additionally, there is a shift in the parameters \gamma and \delta between the two GM scenarios in the sequential setting, any idea why? I would recommend to plot median values instead.

*We believe that when the number of points is not too high, it is generally more informative to show individual points rather than a summary like the median. The difference for beta_kill is indeed not large but it is a significant difference (p=0.0005 by a t-test). Gamma and delta also show biases, and indeed in the opposite direction depending on which dataset is used first. We’re not sure why this is the case. Perhaps it has to do with having slightly less measurements for the lynxes than for the hares (for the hares there is also information on the natality). With the gamma and delta parameters being more directly related to the lynxes they might show a dependency on the order of sequential inference (even though the parameters are all coupled).*

- Line 372: typo "feecback" → "feedback"

*Thanks for spotting this; we fixed it.*

- Second model: All the cell lines have the same parameter values? Was the data normalized? Did the authors use log or natural parameters? As Fig. 9E shows negative values for parameter I would assume is log, but is not clear in the text.

*We thank the reviewer for pointing this out. The parameters are indeed the same for all cell lines (note that they are scalar variables, whereas the activities are vectors). We indeed failed to mention that some of the parameters are estimated on a log-10 scale, we added a sentence to describe this. The data was indeed normalized, between 0 and 1. The entire reasoning behind this model is quite involved, so for further details about the model we refer the reviewer and reader to the paper in which the model is described (References 5 and 36).*

- Figure 1: Axis labels are missing for some of the subplots. I recommend using subsections (e.g., A-B-C) and refer to what is seen directly in the caption. The caption is rather generic and I have difficulties to follow the content of the image. Moreover, a legend will help as, e.g., red solid line and/or black dashed line, are not defined. I do not know what is the difference between the middle column and the right column subplots.

*We apologize for the lack of clarity.* *We added axis values and additional clarification in the figure and in the caption.*

- Figure 2: Axis labels are missing, something like \theta_1 and \theta_2 (instead of the model parameters for simplicity). In the manuscript kernel density is shortened as "KD" while in the figure is shown as "KDE". Same for Gaussian mixtures ("GM" vs "GMM"). The authors comment that "slight differences ... can be observed as well" maybe can be highlighted? It is difficult to spot.

*We’ve added labels at the top and corrected the abbreviations to be consistent with the text. The differences are indeed slight, but you can observe, for example, in the truncated GM that there is a slight clustering of points near the (1.0;0.0) area which is not present in original samples.*

- Legend Fig. 4: "posterior predictive" → "posterior prediction"

*We believe the term “posterior predictive” is actually right here; it is a term used more often for the distribution of new data conditional on the observed data and the model (see e.g. Gelman et al, Posterior predictive assessment of model fitness via realized discrepancies, Stat Sin 1996).*

- Legend Fig. 4: It is unclear for me why the authors normalize again with the maximum. The data are already relative. If the normalization is used, I think that it would be important to clarify how the simulations are normalized. Also with the measured maximum, or with the simulated maximum?

*We apologize for the confusion; the data is not normalized again. The sentence in the legend was just meant to emphasize that we are doing inference against the normalized data. But seeing that it can lead to confusion, we removed it from the legend, and instead mention in the text that the original data is normalized by dividing by the maximum observed value.*

- Fig. 5: As the choice of the prior is often random, it would be interesting to see the effect of the prior, in particular what happens if the prior is less informative.

*In this case using non-informative/broader priors results in unrealistic situations as the model will then prefer to oscillate exactly through the data points with very high frequency. That gives a perfect data fit but requires unrealistically high birth rates, for example. The prior was therefore deliberately chosen to prevent this (as mentioned in the text), so that it only gives realistic fits.*

- Fig. 5 (D): This figure is not completely clear for me. How was the comparison performed? Furthermore, I was wondering why the correlations always increase with sample size but the RMSE does not necessarily decrease?

*We changed this figure to just show the accuracy with a fixed number of samples (1,000), in line with changes to figure 3. But the RMSE and correlation can indeed show (slightly) different trends. The correlation is estimated using samples that were drawn from the target distribution. Hence if the approximation contains areas with high density which are not present in the target, the correlation at the samples can still be very good; however in that case the RMSE will increase as the density at the location of the target samples is lower than it should be.*

- Caption for Fig. 5: There is a extra "3." in the end.

*Thanks for noticing this; it has been removed.*

- Fig 7 (A) comparison of the results with the log10 shown in fig. 5 is difficult, I would suggest to plot in log10 the X axis for a direct comparison and clarify in the caption that the sampling was performed in linear scale.

*We think it is better to show the regular scale in Figure 7 to emphasize that this is a different situation than in Figure 5, namely that we are now doing inference with bounded priors.*

- Fig. 8: Are the mean and standard deviation values calculated after removing burn-in phase?

*Yes; we added a sentence to the methods section to clarify that we always remove a burn-in period and do not adapt the proposals after the burn-in period.*

- Caption for Fig. 9 (E) definition for the gray dash lines are missing, I assume that it corresponds to the results from the individual fittings (untreated / treated).

*Indeed; we added this missing description to the caption.*
